# Public and Healthcare Professional Attitudes Towards Risk‐Stratified Bowel Screening: A Qualitative Study Using an Info‐Comic Book

**DOI:** 10.1111/hex.70315

**Published:** 2025-07-04

**Authors:** Hannah Miles, Una Macleod, David Weller, Joanne Cairns

**Affiliations:** ^1^ Hull York Medical School University of Hull Hull UK; ^2^ University of Edinburgh Edinburgh UK

**Keywords:** acceptability, bowel cancer, bowel screening, colorectal cancer, info‐comic book, qualitative, risk stratification, visual methods

## Abstract

**Background:**

Screening for bowel cancer (colorectal cancer, CRC) is well established in many high‐income countries. There has been considerable interest in moving towards risk‐based bowel screening to increase the efficiency and effectiveness of screening. This UK‐based qualitative study explored public and healthcare professionals (HCPs)' attitudes towards risk‐based bowel screening.

**Methods:**

Five virtual focus groups were held with members of the public of bowel screening age (60–74 in England; 50–74 in Scotland) and HCPs to explore attitudes towards risk‐based bowel screening. Public participants (*n* = 12) were invited through our existing patient and public involvement (PPI) networks. HCPs (*n* = 11) were recruited through existing networks and with the support of screening hubs.

A co‐created info‐comic book was used to facilitate discussion on bowel cancer risk factors. Following transcription, qualitative data were analysed thematically.

**Results:**

There was consensus that more intense screening for those of higher risk is acceptable, but this should not imply less screening for those of lower risk. There was some agreement between the public and HCPs over concerns with undue focus on risk factors, which could disadvantage those with minimal risk factors. There was also a desire to streamline existing bowel screening across the UK nations. It was felt that the current screening programme, by treating people with all risk levels in the same way, is equitable—so clear communication is needed if this is to be changed.

**Conclusion:**

Findings indicate a preference that any changes to the bowel screening programme should enhance the current screening offer, and not compromise screening offered to individuals deemed to be low risk. Changes need to be acceptable to the public and HCPs—if unacceptable, there is a risk of lowering bowel screening uptake, which could potentially exacerbate health inequities in screening outcomes.

**Patient and Public Contribution:**

The info‐comic book was co‐created with two PPI networks, INVOLVE Hull and People and Research Together, Bowel Research UK, supported by Humber All Nations Alliance. The PPI network provided invaluable feedback on the development of the info‐comic book, to ensure inclusivity and avoid the reproduction of dominant stereotypes associated with bowel cancer.

## 1 Introduction

Bowel cancer is the fourth most common cancer in the United Kingdom with approximately 44,000 people diagnosed annually [1], but it is the United Kingdom's second largest cancer killer, with over 55% of bowel cancers detected at Stages 3 or 4 [2]. If detected at Stage 1, there is a 90% 5‐year survival rate, compared to just 10% when detected at Stage 4 [3].

The purpose of the Bowel Cancer Screening Programme (BCSP) is to detect bowel cancer at an earlier, more treatable stage. The BCSP currently stratifies by age, with screening in England being offered to everyone between the ages of 60 and 74, although the age range is currently being lowered to 50–74 years, in line with Scotland. Screening frequency is every 2 years, and the screening test is completed at home and is then sent for testing. If the result is positive, a colonoscopy is offered, which checks for polyps and cancer. The 2022/23 coverage^1^ rate was 72%, an increase on the previous year 2020/21, 68.9% [4]. The switch from the more burdensome Faecal Occult Blood Test (FOBT) has improved accessibility with the newer Faecal Immunochemical Test (FIT) home test requiring just one sample.

There have been growing calls for cancer screening to become risk‐stratified [5]; this transition appears to have public support [6, 7, 8, 9], at least in principle. Risk‐stratified screening would involve moving away from a ‘one‐size‐fits‐all’ model to a more personalised approach, which considers modifiable (lifestyle) and non‐modifiable (genetic predisposition, polygenic risk and family history) risk factors beyond age alone. Stratified approaches involve providing more frequent and/or intensive screening to those at higher risk—and, potentially, less frequent and/or intensive screening to lower risk individuals. Hypothetically, this could involve repeat FITs, for instance, or colonoscopy referral in place of FIT. In this way, it could potentially deliver improved outcomes without extra cost [10].

Compared to breast and cervical cancer, research on risk‐stratified bowel screening has only recently begun to receive attention in the United Kingdom despite some countries already piloting risk‐based approaches or having well‐established guidance for screening programmes; for instance, in the case of family history of bowel cancer, as shown in our international scoping review [11]. Quantitative FIT (qFIT) can be used to stratify screening pathways as opposed to a simplified ‘positive’ or ‘negative’ result based on a fixed threshold, currently 10 µg/g among symptomatic patients and between 80/120 µg/g among asymptomatic (BCSP) in England and Scotland, respectively.

The risk of developing bowel cancer varies considerably between individuals depending on various factors such as age, sex, lifestyle and genetics. Increased risk is associated with inflammatory bowel disease, previous bowel cancer history in first‐degree relatives, body mass index (BMI), smoking history and diet, particularly the consumption of processed and red meat [12].

A major concern with any change to the existing BCSP is the potential impact on uptake, so it will be crucial to demonstrate whether stratified screening would impact uptake or reinforce existing socio‐economic disparities. Although uptake has significantly increased since the implementation of the FIT [4], uptake remains lower among those experiencing greater socio‐economic disadvantage [13], and minoritised ethnic groups [14, 15, 16, 17]. Therefore, any proposed changes to the current BCSP need to be acceptable, both to those delivering and receiving bowel screening. Changes that lack acceptability could be counterproductive and risk lowering uptake further, potentially widening pre‐existing socio‐economic and ethnic screening inequalities. For instance, those deemed to be higher risk may not be willing to undergo more intensive or frequent screening, whereas those deemed to be lower risk may not feel comfortable with de‐escalated screening, although it is more likely to be the latter based on previously published studies [8].

This paper presents the findings from an exploratory qualitative study, which explored public and healthcare professionals (HCPs)' attitudes towards the acceptability of risk‐stratified bowel screening.

We posed the following research questions:

1. *How acceptable is risk‐stratified bowel screening to those eligible for bowel screening?*
2. *How acceptable is risk‐stratified bowel screening to HCPs?*

## 2 Materials and Methods

The full protocol is registered with the Open Science Framework [18]. The data discussed in this article were produced from an exploratory qualitative study using virtual focus groups, supported by a visual elicitation tool, in the form of an info‐comic book. The development of the info‐comic book is described in full elsewhere [19].

### 2.1 Participants and Recruitment

Purposive sampling was used to select and recruit participants. Purposive sampling enables the selection of participants who are especially knowledgeable about, or experienced with, the subject matter [19], in this case, those eligible for screening and HCPs who work in the BCSP. As this was a modest sample, stratified purposeful sampling techniques helped ensure that there was representation and diversity within the sample population, by capturing variations—age, sex, geographical location and ethnicity. In addition, we included various professional roles within the BCSP, so a range of voices could be captured across the screening programme. Snowball sampling was used to contact potential participants through existing personal and professional networks. Combining sampling techniques helped ensure that a range of participants were identified and invited to take part. The final sample included 12 public participants and 11 HCPs/allied stakeholders.

### 2.2 Recruitment of Public Participants

Participants were invited through existing patient and public involvement (PPI) networks: INVOLVE Hull, People and Research Together and Bowel Research UK. The inclusion criteria required participants to be of BCSP screening age and to have received their first bowel screening invitation. This ensured that participants had some knowledge of the screening programme and how the invitation process works. However, participants were not required to have accepted the invitation for screening—we considered it was equally important to include participants who chose not to participate in screening. A brief questionnaire was created using JISC Surveys to screen potential participants for eligibility and aid our sampling.

### 2.3 Recruitment of HCPs

HCPs were recruited through existing networks and with the support of screening hubs, who sent details of the research out to their networks. The inclusion criteria stated that any HCP or allied stakeholder, for example, screening programme managers, could participate if they worked in a role associated with the BCSP. The final group of participants came from a diverse range of professional backgrounds, including specialised screening practitioners, screening hub managers, allied screening hub staff and HCPs from primary and secondary care.

### 2.4 Virtual Focus Groups

Five online virtual focus groups were held using the online conferencing platforms: Zoom and Microsoft Teams. Two researchers (H.M. and J.C.) facilitated all five focus groups, which lasted approximately 60 min. Two focus groups were held with members of the public of bowel screening age (*n* = 12). Three focus groups were held with HCPs and allied stakeholders (*n* = 11). The focus group discussion was supported using a visual elicitation tool in the form of an info‐comic. The info‐comic book used fictional characters presenting with a range of risk factors. These risk factors could be assessed as low, moderate and high risk, potentially elevating or lessening their risk to developing bowel cancer (colorectal cancer [CRC]). Please note that these categories were based on hypothetical scenarios based on fictional characters and informed by the risk factors identified in our review [11].

A topic guide was used to facilitate discussion; its development was informed by the scoping review and our previous knowledge of risk‐stratified screening and following input from members of the PPI group. Questions for both members of the public and HCPs involved acceptability and communication of risk, while HCPs were also asked about any implementation challenges to risk stratification.

### 2.5 Visual Elicitation Tool: Info‐Comic Book

We used visual elicitation tools, in the form of an info‐comic book (Box 1). The main purpose of this co‐created info‐comic book [20] was to assist with the dissemination of risk‐based information and as a tool to help facilitate discussion. During focus groups, we asked participants to think and reflect upon the six fictional characters featured in the info‐comic book and their associated risk factors. We grouped the characters into categories based on risk factors known to elevate or decrease one's level of risk of bowel cancer (Supplementary Appendix). For instance, 54% of colorectal cancer are preventable and owing to increased risk factors such as being physically inactive (5%), alcohol consumption (6%), smoking (7%), being overweight or obese (11%), processed meat consumption (13%) and 28% for diets with low fibre intake [1]. Additionally, participants were asked to think about, and discuss, the ‘acceptability’ of any potential changes to the BCSP. In this way, the info‐comic book proved to be a useful ‘third prompt’ [21] as described in Box 2.

Box 1 **Visual elicitation tools—key features.**

- Visual elicitation tools such as infographics, info‐comic books, animation and photo elicitation are becoming increasingly popular, in line with the increase in creative and visual methods in qualitative research [22], which can be more accessible to a diverse range of population groups.
- They can be a supportive tool in the generation of conversation and ideas [23].
- Incorporating visual elicitation tools can be highly useful in enhancing the depth of the data generated [23].
- They can be successfully combined with other qualitative methods, such as focus groups and interviews, and can be successful in engaging under‐reached groups [24].
- They can assist with conveying complex messages, in an accessible and playful manner [25].
- In this study, the info‐comic book helped convey complex health messages visually in a more accessible format.

Box 2 **Use of info‐comic book as ‘third prompt’.**

- A ‘third prompt’ can be an object, or similar, that can be used to enable a critical distance from which discussion can take place.
- This can be particularly useful in scenario‐based research or when discussing difficult ethical decisions about sensitive or contentious research topics. In this study, we found that the info‐comic book meant that participants could use the characters as a tool for discussing sensitive information, shifting the focus from the self.
- We found in this study that the use of an info‐comic book provided this critical distance, and could be used effectively to detract, or steer sensitive research conversations away from individual participants to broader scenarios, whilst still being able to relate to the characters.
- In this way, the info‐comic book then became an important prompt that could be used to facilitate reflection and discussion outside the immediate researcher and participant interaction.

Although visual elicitation tools can be used effectively, they are not without challenges [20, 22]. Visual methods can be viewed as ‘arbitrary’ and ‘subjective’, open to multiple interpretations based on diverse social and cultural environments [26], and the significance and value afforded can be culturally and socially determined. It is also important to reflect upon the role a visual tool will play within the broader methodological approach, and whether they will enhance or detract from the main research objectives. In this study, the info‐comic book's main purpose was to convey complex health messages in a more accessible and easier format.

Incorporating visual elicitation tools like info‐comic books should be a considered process. Even though the info‐comic book used in this study had been designed in collaboration with members of the public and a local artist, we still found that on occasion, key messages were missed or misinterpreted. For instance, one participant became confused with the elevated risk associated with having cancer previously, particularly when the primary and secondary locations of the cancer may differ. This meant that at times, researchers had to verbally explain aspects of the info‐comic book.

### 2.6 Ethics

Full institutional ethical approval was granted for this study. All participants were informed about the study via the participant information sheet and were able to ask any questions they had before taking part. All participants provided written informed consent, and verbal consent at the start of each focus group.

### 2.7 Data Analysis

Focus groups were audio‐recorded, transcribed verbatim and pseudonymised at the point of transcription to assist with anonymity. Focus group data were analysed by drawing upon Braun and Clarke's [27, 28] approach to Thematic Analysis (TA). Braun and Clarke suggest that approaches to TA should be understood as a ‘family of methods’, and the ‘designation of TA as a theoretically flexible method, rather than a theoretically informed and delimited methodology’ [29]. Familiarisation with the data involved a thorough reading of the transcripts, with initial thoughts and reflexive comments. Following this, initial coding of the data conducted by H.M. and J.C. involved categorising data into thoughts, feelings and concerns surrounding the acceptability of changes to the BCSP. This stage followed an inductive data‐driven approach, where initial codes were developed from the data. This enabled a coding of the data by applying both descriptive and analytical concepts and codes, alongside reflexive comments that helped aid the analysis process. However, we also combined this with the use of a codebook, which within TA can be viewed with some trepidation [29]. However, we were mindful that the codebook was developed to support the coding process, rather than a rigid framework onto which other codes must be mapped, or code consensus sought. Rigour in qualitative research is achieved when the study methodology is systematic and transparent through complete, methodical and accurate reporting [30] and the use of researcher reflexivity and rigorous peer review [31]. In addition, applying Lincoln and Guba's [32] concepts of credibility, dependability, confirmability and transferability can assist with the trustworthiness of the findings, and this was achieved in several ways in the study. The protocol went through robust peer review before being submitted for ethical approval. To enhance credibility and confirmability, authors (H.M. and J.C.) met regularly throughout the coding process, sharing analytical ideas and reflexive comments. Although full member‐checking, where participants review transcripts, was not possible within the time limits of this study, credibility and dependability were considered by discussing and sharing preliminary findings with colleagues not involved with the data analysis process, both within and outside of the research team. Although these findings most obviously relate to the acceptability of bowel screening changes, they might prove fruitful when thinking about the acceptability of risk stratification within other screening programmes. We have aimed to assist this process by describing the context and methods, which can assist with transferability. Confirmability was supported by a transparent description of methodological steps taken in the study and through researcher reflexivity. This study adhered to protocol‐driven methods (registered with the Open Science Framework [18]); while analytical reflexive notes were made throughout the data analysis process, these were recorded alongside emerging codes and potential themes.

## 3 Results

### 3.1 Focus Group Participants

Twelve public participants took part, eight identified as female and four as male. All participants were over 60 years of age. Ethnicity was self‐defined and was not a predesigned question. Nine participants described their ethnicity as white British (WB), one African, one white/Asian, one white/black Caribbean. Of this sample, ten had accepted an invitation for screening and had participated in the national BCSP, two had unclear screening status. There was a reasonable geographical spread across England, and one participant from Scotland (Table 1). Eleven HCPs working across England associated with the provision and delivery of the BCSP took part in the focus groups (Table 2).

**Table 1:** Public focus group participants and characteristics.

Participant	Pseudonym	Sex (including transmale/female)	Ethnicity	Region	Accepted national bowel screening invite
Focus Group 1					
Participant 1	Daniel	Male	WB	South East England	Yes
Participant 2	Susan	Female	WB	Yorkshire and Humber	Yes
Participant 3	Paul	Male	WB	Yorkshire and Humber	Yes
Participant 4	Helena	Female	WB	North East England	Yes
Participant 5	Mary	Female	WB	Yorkshire and Humber	Yes
Participant 6	Andrew	Male	WB	Yorkshire and Humber	Yes
Focus Group 2					
Participant 7	Penny	Female	WB	South West England	Yes
Participant 8	Edward	Male	WB	Yorkshire and Humber	Yes
Participant 9	Eleanor	Female	WB	South East England	Yes
Participant 10	Grace	Female	African	London	Yes
Participant 11	Layla	Female	White/black Caribbean	South West England	Unclear *participant did not specify either way
Participant 12	Asher	Female	White/Asian	Scotland	Unclear

**Table 2:** Healthcare professionals' focus group participants and characteristics.

Participant	Occupation/role	Region of United Kingdom
Focus Group 1		
HCP 1	Programme manager	Yorkshire and Humber
HCP 2	Lead specialist screening practitioner (lead SSP)	Yorkshire and Humber
HCP 3	Gastroenterologist	Yorkshire and Humber
HCP 4	GP	Yorkshire and Humber
Focus Group 2		
HCP 5	Lead specialist screening practitioner (lead SSP)	North East England
HCP 6	Specialist screening practitioner (SSP)	North East England
HCP 7	Specialist screening practitioner (SSP)	North East England
HCP 8	Specialist screening practitioner (SSP)	North East England
Focus Group 3		
HCP 9	Nurse consultant	London
HCP 10	Programme manager	London
HCP 11	Service manager	London

The results are organised around four key themes that explore participant attitudes and acceptability towards risk‐stratified bowel screening. The themes identified included the following: attitudes towards risk stratification; communication change; faith in the current screening programme, and HCPs' acceptability towards managing change within the BCSP.

Overall, participants did not reject the notion of risk‐based screening, particularly in relation to more intense screening for those assessed to be of higher risk. However, our results demonstrate that there was some concern that this could mean there would be less screening for those assessed to be lower risk. Both the public and HCPs raised concerns over focusing on identifiable risk factors, for instance, public participants relayed examples of friends or relatives who had been diagnosed with cancer but had not presented with any particular risk factor—at least not one that could be readily identified. Participants discussed having faith in the current BCSP, and stratifying by age was equitable. HCPs felt that resources could be better focused on streamlining what is already in place. Therefore, any potential changes to the BCSP must be communicated effectively and sensitively.

#### 3.1.1 Theme 1: Attitudes Towards Risk Stratification in the BCSP

Participants expressed concern with stratifying the BCSP by additional risk factors, other than age. For the main part, there was concurrence between public participants and HCPs' views on this; both the public and HCPs drew upon a similar narrative that centred on the often‐unpredictable nature of cancer. In relation to risk stratification, this raised some concerns amongst participants by placing everything in what Andrew referred to as a ‘*risk‐based basket’*:

You know, sometimes someone who seems incredibly well suddenly get stricken down by a severe illness and it's a matter of great surprise to other people. So, you sort of, say, well, how did that happen? Because you know they kept themselves so well. So, I'm not sure about putting all your eggs in the ‘risk‐based basket’.

(Andrew, age 74)

Public participants often drew upon personal experience to explain and justify their concerns. For example, knowing someone who had been assessed as lower risk but had gone on to develop a cancer. Moreover, Andrew is referring to the idea that if we work from the premise that everyone has an identifiable risk factor before developing a cancer, then what, or whom, do we risk overlooking? For example, Eleanor felt this was just looking at ‘part of the problem’:

I think that's just looking at part of the problem, I mean I know so many people who have led exemplary healthy lives never smoked, don't drink, practice yoga all their lives, and come down with terrible cancers.

(Eleanor, age 72)

And Eleanor went on to say:

I don't know whether you should be trying to differentiate between risk factors, or just try and screen as many people as you possibly can, and maybe bring the age down […] then you're not differentiating, and then you are not sort of targeting certain groups, everybody has the opportunity.

(Eleanor, age 72)

These concerns could potentially impact the level of acceptability that the public has towards more risk‐stratified screening approaches. For example, some of the public participants raised concerns that risk stratification could mean that those who were assessed as lower risk might be ‘forgotten about’. Daniel felt he would be assessed as lower risk; therefore, would he be potentially marginalised within this process:

So, I think one needs to be very wary of just taking off the cuff comments that says, ‘high risk’ ‐ it's obviously good to do that group, and I personally would say I'm in a lower risk group therefore ‘forget about me’, because that sod's law can really come through to kill you.

(Daniel, age 66)

Conversely, Mary was concerned that it could cause complacency amongst those assessed as lower risk:

If they are taking responsibility for their health it could lead to complacency and think, you know ‘I'm doing everything right, I'll be fine’ and it doesn't work like that.

(Mary, age 67)

HCPs demonstrated similar concerns; however, as expected, these centred on the challenges that modifying the BCSP by risk factors could present for clinical practice; particularly around identifying symptoms and wider diagnosis, as it assumes that everyone presents with an identifiable risk factor beyond age.

I think it's challenging, I think partly because one of the ideas that we're assuming is that everybody with a bowel cancer must have some kind of risk factor.

(HCP3, gastroenterologist)

Similarly, you know, cancer occurs because of mutations, and random mutations. We have to be so careful that, yeah, there are certain things that we can identify as risk, but for X amount […] they won't have any of those risks, and I think that is really something, we have to be so careful that you don't rely on a risk stratification necessarily.

(HCP4, GP)

And

So, you can be the fittest, healthiest person with a BMI of 21. Never smoked, don't drink alcohol or sugar, and have a very nasty tumour.

(HCP9, nurse consultant)

This HCP's sentiment echoes Eleanor's previous argument relating to potentially overlooking bowel cancer risk for those who may be at lower risk, due to adopting healthy lifestyle behaviours.

#### 3.1.2 Theme 2: Attitudes Towards Communicating Risk Factors and Change in the BCSP

There was general concurrence between the public participants that any changes to the BCSP must be communicated clearly, otherwise changes could exacerbate existing inequalities in access and take‐up of the screening programme. For example, Edward raised concerns that it could mean that overall fewer people would be screened:

But I do feel that to try and focus it [screening] might lead to fewer people being screened, even though they might be slightly higher risk. I mean I'm generally in favour of the more the screening the better.

(Edward, age 70)

Any changes could be viewed with suspicion, such as, being a politically motivated money‐saving agenda:

The concern is that it's seen as a means of saving money rather than saving lives, I'm sure the government would like to pare back, or some governments might want to pare back screening generally.

(Edward, age 70)

Grace raised an important point from the perspective of someone who might be considered highrisk, having had cancer previously. For Grace, it was imperative that risk and any associated changes are explained in a clear and supportive way, otherwise fear may prevent people from attending:

…so, I don't know how you're going to make this, you know not as scary. Because that's why people don't even go in the first instance. How do we start the conversation that ‘this is going to happen, this is what to expect’. Because sometimes it's the fear, like I had cancer twice and it's just the unknown, like, ‘oh my God what?’.

(Grace, age 60)

Relying solely on risk factors to stratify a screening programme raised concerns for both public participants and HCPs working within the programme. These concerns centred on the problems potentially associated with identifiable risk factors, and who, or what this could overlook.

Public participants also raised concerns over low risk creating misplaced feelings of ease and complacency. These concerns highlight that any changes made to the BCSP need to be communicated sensitively and carefully. Communication needs to ensure that those classified as lower risk do not feel as if they are being marginalised or forgotten about through these changes, and that guidance over risk factors is clear. The narratives presented perhaps hint at a subtle concern that a screening programme based purely on risk stratification could remove some of the nuances involved in decisions over screening participation. Comments made by both the public and HCPs demonstrate concerns with regard to the limitations created by relying solely on identifiable risk factors.

#### 3.1.3 Theme 3: ‘If It's Not Broken…’: Faith in the Current Screening Programme

Participants had not only faith but also sought comfort from the present age‐stratified BCSP. Both HCPs and public participants felt it was both fair and equitable, enabling everyone to access the programme, if they chose to:

This is what the beauty of an age stratification is. It allows everybody to be involved, whether or not they've had the family history.

(HCP3, gastroenterologist)

The current programme was viewed as providing ‘blanket cover’ and that this ‘feels equal’. This raised concerns around whether a programme based on risk factors would remove that equitability:

I would just say that there is something to say about the current screening programme that it has that blanket cover, […] it just captures people, and you know, we do that, obviously in that age category.

(HCP4, GP)

And

I just, I mean, I suppose, for me the screening is covering a general population, isn't it? And to me it, sort of… feels equal, that everybody gets that kit through the door, it's up to you whether you want to fill it in or not.

(HCP 7, specialist screening practitioner [SSP])

Public participants also discussed having faith in, and reassurance from, the current screening programme. The current programme also removes some element of responsibility and accountability; for example, the need to recall family history.

Andrew describes feeling reassured by what he refers to a ‘periodic check‐up’:

Personally, I've always felt reassured that there is this sort of, you know, periodic check‐up.

(Andrew, age 74)

Penny returns to the previous narrative surrounding not knowing what everyone's risk factors are. However, she also supports this by describing the reassurance she gets from the current screening programme stratifying by age only, so there is no differentiation between identifiable risk factors and therefore people accessing the programme:

My sort of idea is that when you get to a certain age, say 60 onwards, you have the screening, and there's no sort of differentiation there between the risk factors. We don't know what the risk factors of all 60‐year‐olds are, so everybody 60 onwards get screened.

(Penny, age 73)

In the narrative below, Mary makes an important point surrounding decision‐making, which raises potential concerns over accountability and responsibility, and where the authority over such decisions will ultimately lie:

I mean everybody is screened at 60 now and has the opportunity to be screened at 60 now, if it was based on risk, who has the responsibility to call it, is it down to a person or to a professional.

(Mary, age 67)

The way in which changes to the BCSP are communicated appeared to be key for all participants, otherwise they identified the potential for some people to feel as if looking after their health has unwanted consequences; for example, being screened less often. This concern is captured in Edward's narrative below, where he explains that although he is not against prioritising those deemed higher risk, but that this should not be at the expense of the current programme, or what people are currently able to access:

Yes, I'm not against prioritisation, but I wouldn't want to lose the continuous process of screening people over a wide age band, as we have now. Um, or even extending it. But I mean if people do have higher risk factors, then I'm not against them as an increase in service being prioritised.

(Edward, age 70)

#### 3.1.4 Theme 4: Streamlining: Workforce and Resource Challenges

HCPs were concerned that the addition of further levels of risk stratification would increase pressure on what they felt was an already overstretched service. Two of the main issues that HCPs highlighted were the current workforce gaps within the BCSP and the lack of available resources, including equipment, facilities, time and capacity issues. Subsequently, HCPs suggested that streamlining what is already in place would be a better use of time and resources.

For example, HCPs highlighted issues with current workforce shortages:

[…] accredited colonoscopies are like gold dust… most centres are struggling to recruit. We had the rolling job advert out for six months, and we didn't have one taker.

(HCP1, programme manager)

And the amount of time it takes to train clinical staff:

These staff don't just appear, it takes years and years to train them and getting them up to, you know, a suitable level.

(HCP5, lead SSP)

HCPs also drew attention to the current workforce challenges that go beyond the BCSP, noting that these also have a significant impact on what can be offered:

There is a massive workforce issue within England, we have a large number of vacancies for a nursing workforce […] if you expand any service you need to be able to have the workforce to do it, and of course the equipment and the facilities.

(HCP10, clinical programme manager)

[…] we've got a staffing crisis anyway in the NHS. Adding this in [risk stratification] isn't really gonna help, we need to kind of streamline stuff rather than adding extra services in and extra steps.

(HCP5, lead SSP)

HCP5 notes that their preference would be to streamline what is already in place, and they were not alone with this observation. Other HCPs also felt that at this current time, it would be a better use of resources to focus on developing and improving the current screening programme. This is in addition to making changes that bring it in line with what is offered in other parts of the United Kingdom, like Scotland, where both the screening age and FIT thresholds^2^ have been lowered.

I think what we'd be better to do […] is to be in line with Scotland and get the FIT to 80 […] having access at 50 to 74, which I think would be the most effective thing […] that would be much more effective, and a better use of our time […] and would probably save far more people.

(HCP9, nurse consultant)

Similarly, a clinical programme manager said:

[…] we should be looking at what we're doing across our Four Nations […] can we bring our FIT to 80 […] and making sure that we're screening between 50 and 70.

(HCP10, clinical programme manager)

One of the public participants also felt that it would be more effective, and equitable to lower the age range and focus on screening more people:

[…] as previously mentioned, the age range should be adjusted.

(Layla, age 64)

An important issue raised was the association between lifestyle factors and stigma. For example, could risk stratification further elevate the stigma associated with certain lifestyle behaviours.

[…] I think risk stratification could be a really excellent tool, but it has to be done very, very carefully and it's got to be done in a non‐judgmental way …it's got to be done in addition to and develop on what's already in place. So, the two must and need to go together.

(HCP9, nurse consultant)

Subsequently, one strategy proposed by HCPs was to risk stratify people once people enter the BCSP; this might also mitigate some of the issues associated with stigma and access:

I think if we were going to risk stratify, I'd feel more comfortable doing it once they're in the programme I think it would be useful because not everybody goes ahead with screening, so they might engage with us.

(HCP2, lead SSP)

There was a consensus among HCPs that it would be more acceptable to focus on developing the existing BCSP, rather than adding new steps to it as would be required as part of risk stratification, which could potentially add more pressure to an already overstretched service. It is important that any changes to the BCSP do not exacerbate existing health inequalities or create additional barriers surrounding stigma and access. This is in line with some of the points raised in Theme 2, where public participants highlighted the need to ensure that any changes to the BCSP are communicated clearly and sensitively.

## 4 Discussion

Overall, the findings indicate that although public participants did not reject the principle of risk‐stratified screening, they felt that this should not be at the expense of those who are assessed as being of lower risk. Our data revealed concerns over the acceptability of placing too much emphasis on identifiable risk factors. Generally, public participants agreed that the current status quo in screening (stratification by age) is preferable as it is equitable, since low risk does not necessarily mean no risk. However, they were prepared to accept more frequent or intense screening regimes for those who were assessed as higher risk. This is also echoed in other relevant research [8, 33] where most highrisk individuals were more likely to take up screening, whereas low‐risk individuals were less supportive of reduced screening frequency. Some participants indicated that more screening is preferable; however, with the risks associated with over‐screening, particularly the over‐reliance of colonoscopies as seen in certain countries, could be counter intuitive. Moreover, there are resource constraints, as indicated by HCPs when discussing the accreditation process involved in training staff to be able to effectively undertake colonoscopies.

HCPs also raised concerns over adopting a purely risk‐stratified approach, arguing instead for a concerted effort being directed at developing the current screening programme. For example, increased acceptability would include considering sex differentiation, implementing a FIT cut from 80 µg of haemoglobin per gram of stool (µg/g), currently 120 µg/g in England, alongside expanding the screening age from 50 to 74 years in line with Scotland. Although there did appear to be different perceptions amongst some HCPs of what a risk‐stratified screening programme could possibly entail, demonstrating the need for clear communication and guidance surrounding any changes being made to the screening programme, as suggested elsewhere [34], and the potential role of HCPs in communicating this to those offered different or reduced screening [35].

Our findings correspond with similar research conducted on public acceptability of risk‐stratified bowel screening, whereby members of the public are in favour of being offered more, but not less screening [36]; therefore, this viewpoint should be carefully considered before altering existing screening programmes. Dennison et al. [37] conducted online community juries with a stratified sample. Results showed that while generally risk stratification was acceptable, there were concerns regarding the fair implementation of this, akin to our findings surrounding equity. Therefore, it is imperative that equity issues are considered before making any modifications to the BCSP to ensure we do not further widen pre‐existing inequalities in uptake.

## 5 Strengths and Limitations

Strengths of our study include its protocol‐driven methods; the involvement of PPI from inception; using the same tool in both the patient and HCP focus groups helped to focus the discussions by presenting hypothetical scenarios in relatable ways to both sample groups; the benefit of visual methods to help convey what can be both complex and sensitive health messages, and the alignment with published results elsewhere. Nevertheless, it was a relatively small‐scale study that mostly involved those who are already engaged with the screening programme and as such participation is likely to be highly associated with attitudes to screening. Further, there are challenges in designing visual elicitation tools which are perceived equally by all. However, a main goal of using these tools in research is to enable wider and more equal participation. One strategy could be to ensure that a good level of social and cultural diversity is achieved across patient and public consultation groups. Therefore, ensuring that a range of views is captured and considered when designing visual tools.

## 6 Conclusion

Caution should be exercised when making changes to bowel screening, and any changes need to be communicated effectively and sensitively, otherwise there is a risk that there will be resistance from screening invitees, based on the views captured in this study. However, this study is limited because we are asking about attitudes towards risk‐stratified bowel screening based on hypothetical, not actual risk. Future research should seek to address these uncertainties by exploring attitudes towards risk‐stratified screening, when individuals are offered alternative risk‐stratified screening, to see whether these views hold. Research should also explore the potential impact of risk‐stratified screening on health inequities to avoid unintended consequences.

## Author Contributions

**Hannah Miles:** methodology, investigation, writing – original draft, writing – review and editing, project administration, formal analysis, data curation, validation. **Una Macleod:** investigation, writing – original draft, writing – review and editing, supervision, funding acquisition. **David Weller:** investigation, writing – original draft, supervision, writing – review and editing, funding acquisition. **Joanne Cairns:** conceptualization, investigation, funding acquisition, writing – original draft, methodology, validation, visualization, writing – review and editing, formal analysis, project administration, data curation, supervision, resources.

## Disclosure

The funder had no role in the design of the study including data collection, analysis, and interpretation of the data.

## Ethics Statement

The study was approved and ratified at the Hull York Medical School Ethics Committee (REC 21/22 31).

## Consent

All participants were informed about the study both verbally and via a participant information sheet. All participants provided full written informed consent, including ongoing/continuous verbal consent at the start of each focus group interview.

## Conflicts of Interest

The authors declare no conflicts of interest.

## Supporting information

Caption for Appendix Figure: Extract from info‐comic book, ‘Did you know your poo could save you?’

## Data Availability

Pseudonymised transcripts are available on request by contacting the corresponding author.

## Endnotes

1 ‘Coverage’ refers to the number of people screened within a set time period out of the total number of eligible people, whereas ‘uptake’ is the number of people who attend screening within 6 months of being invited out of the total number invited.

2 A FIT is a test used in bowel cancer screening to detect invisible traces of blood in the faeces. Different thresholds are used to determine whether someone is offered a follow‐up test, a colonoscopy. In England, the threshold is set at 120 µg Hb/g faeces, whereas in Scotland it is set at 80 µg Hb/g faeces.
