# Supplementary figures and images for: Public and Healthcare Professional Attitudes Towards Risk‐Stratified Bowel Screening: A Qualitative Study Using an Info‐Comic Book
Source: Health Expect. 2025 Jul 4;28(4):e70315. doi: 10.1111/hex.70315 (PMC12227016; doi:10.1111/hex.70315)

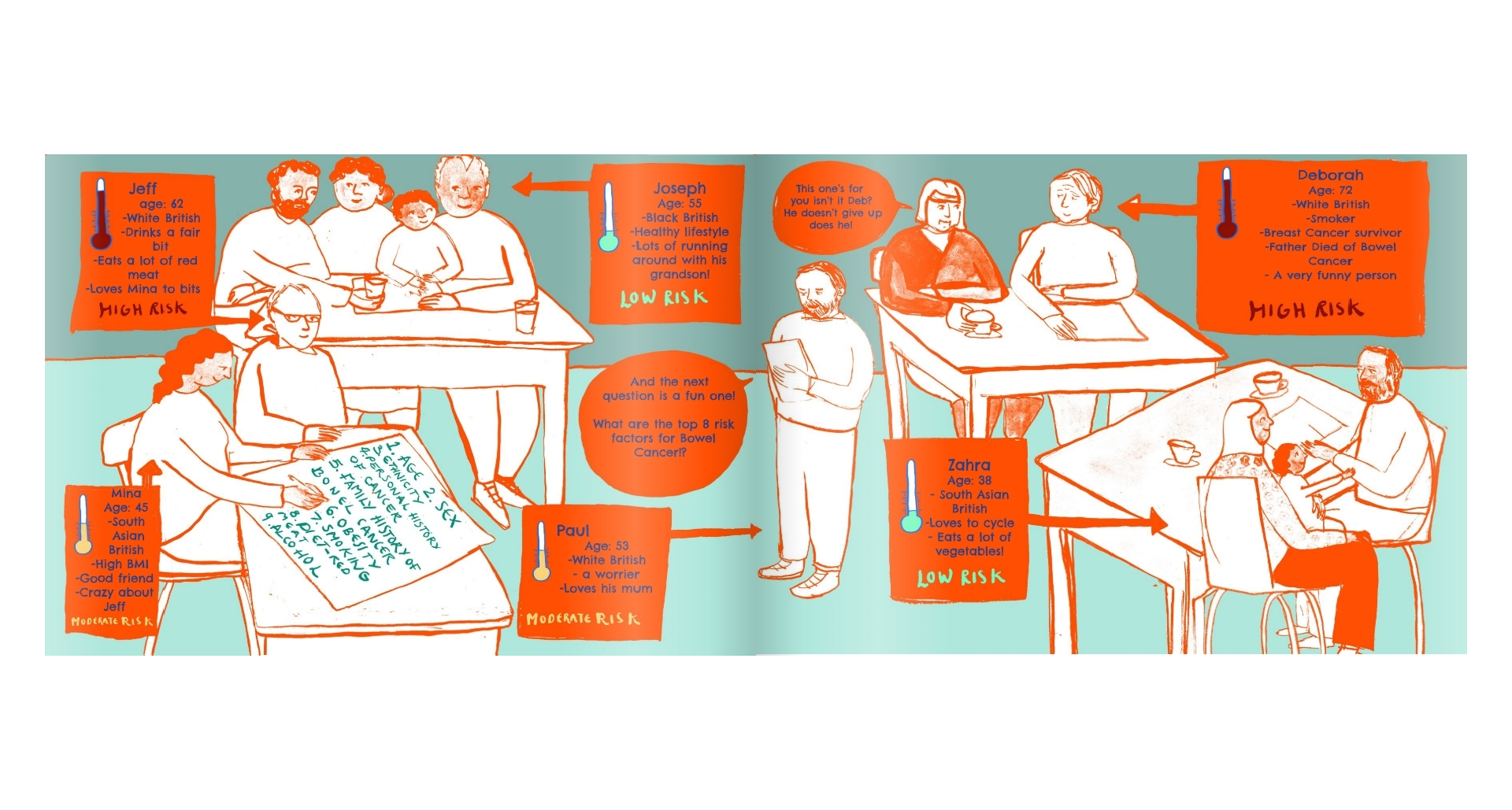

Supplement: Supplementary file 1 — Caption for Appendix Figure: Extract from info‐comic book, ‘Did you know your poo could save you?’ [file HEX-28-e70315-s001.png]
